# Make Intelligent of Gastric Cancer Diagnosis Error in Qazvin’s Medical Centers: Using Data Mining Method

**DOI:** 10.31557/APJCP.2019.20.9.2607

**Published:** 2019

**Authors:** Asghar Mortezagholi, Omid Khosravizadeh, Mohammad Bagher Menhaj, Younes Shafigh, Rohollah Kalhor

**Affiliations:** 1 *Department of Computer Engineering, Islamic Azad University of Qazvin (QIAU),*; 2 *Social Determinants of Health Research Center,*; 4 *General Surgeon, Assistant Professor. School of Medicine, Qazvin University of Medical Sciences, Qazvin,*; 3 *Department of Electrical Engineering, Amirkabir University of Technology, Tehran, Iran.*

**Keywords:** Gastric cancer, risk factors, early diagnosis, data mining, artificial intelligence, machine learning

## Abstract

**Objective:**

Gastric cancer is one of the most common types of cancers, which will result in irreparable harm in the case of misdiagnosis or late diagnosis. The purpose of this study is to investigate the capability of data mining techniques and disease risk factor characteristics to predict and diagnose the gastric cancer.

**Methods:**

In this retrospective descriptive-analytic study, we selected 405 samples from two groups of patient and healthy participants. A total of 11 characteristics and risk factors were examined. we used four Machine learning methods, Include support vector machine (SVM), decision tree (DT), naive Bayesian model, and k nearest neighborhood (KNN) to classify the patients with gastric cancer. The evaluation criteria to investigate the model on the database of patients with gastric cancer included Recall, Precision, F-score, and Accuracy. Data was analyzed using MATLAB® software, version 3.2 (Mathworks Inc., Natick, MA, USA).

**Results:**

Based on the results achieved from the evaluation of four methods, the accuracy rates of SVM, DT, naive Bayesian model, and KNN algorithms were 90.08, 87.89, 87.60, and 87.60 percent, respectively. The findings showed that the highest level of F-Score was related to the SVM (91.99); whereas, the lowest rate was associated with the KNN algorithm (87.17).

**Conclusion:**

According to the findings, the SVM algorithm showed the best results in classification of Test samples. So, this intelligent system can be used as a physician assistant in medical education hospitals, where the diagnosis processes are performed by medical students.

## Introduction

Cancer is a disease in which the cells proliferate abnormally and uncontrollably and can involve their adjacent tissues. Normal cells grow and divide naturally, but cancer cells reproduce and diverge from the natural cell masses. Although different types of cancers have been identified, out of control growth of cells is common among them(de Castro et al., 2018; Kimet al., 2014). Gastric cancer is one of the most common types of cancers, but its diagnosis is difficult in the early stages because it lacks special signs and symptoms. However, scientists have identified some risk factors that predispose a person to gastric cancer. Some of these factors include bacterial infection (Bartfeld et al., 2015), gender, age, race, environment and location, diet (Shimada et al., 2018), previous surgery on the gastric, Pernicious anemia, menetrier disease, and blood type factors(Alkebsi et al., 2018). Although the global prevalence of gastric cancer is decreasing, the prevalence rate of this disease is still high in Asian countries (Zhu et al., 2013). This cancer is more common among people over 40 years (Billiar et al., 2009) and its prevalence in men is two times higher than women(Afshar et al., 2009). Gastric cancer is caused by malignant growth of the gastric cells, which can spread in any part of the stomach or even other organs, especially esophagus, lungs, and liver. Gastric cancer is the fourth most common cancer in the world after lung, breast, and intestinal cancers (Biglarian et al., 2011) and is generally ranked as the second cause of cancer death worldwide (Billiar et al., 2009). Unfortunately, this cancer should reach its advanced levels to show the symptoms such as: 1. Unexplained and unintentional weight loss, 2. Anorexia, 3. Abdominal pain in the stomach area, 4. Feeling early satiety just under the chest after eating very low amount of food, 5. Heartburn, dyspepsia, or symptoms like gastric ulcer, 6- Nausea, 7. Vomiting with or without blood, 8- Swelling or fluid accumulation in the body. Methods such as biopsy, endoscopy, ultrasound, CT scan, X-ray radiography, and special clinical tests are used for diagnosis of gastric cancer (Kim et al., 2014). Considering the unsolvable or not easily solvable issues, a continuous movement has been developed from purely theoretical research to applied research in recent years, especially in the field of information processing. In this regard, researchers showed an increasing interest in the theoretical development of model-free intelligent dynamic systems based on the empirical data. Artificial neural networks are part of these dynamic systems, which process the experimental information and transfer the knowledge or law beyond the data to the system (Menhaj, 1998). Data mining examines and analyzes the databases and massive collections of data in order to discover and extract knowledge systemically (and semi- systemically). Data mining is actually used to solve problems with no or very complex algorithmic solutions, such as the problems related to clinical diagnosis, analysis of medical images, and survival prediction. It is also applicable in a wide range of medical fields including oncology, cardiology and hematology, intensive care, diagnosis from medical images, infertility, surgery, etc (Kurt et al., 2008). Therefore, considering the advancement of machine learning algorithms and the difficulty of diagnosing the gastric cancer by clinical and pharmacological parameters, we need to use computer systems such as data mining more than ever. In this study, we aimed to classify the patients with gastric cancer using the learning methods of support vector machine (SVM), decision tree (DT), naive Bayesian model, and k nearest neighborhood (KNN). This study has been done to determine the possibility of predicting gastric cancer by using data mining techniques and risk factors.

## Materials and Methods 

This applied research was conducted based on the retrospective descriptive-analytic method among the individuals who referred to the selected health centers of Qazvin city in 2017. There are various ways to execution of data mining projects. One of the most powerful ways for implementing of data mining is CRISP. In this paper, the proposed model is based on CRISP, which consists of six phases. Each of these phases itself consists of sub-sections. The forward and backward movement of different phases is required under the input of each phase to the output of the previous phase. These six phases include: Business understanding, Data understanding, Data preparation, Modeling, Evaluation and Deployment.

**Table 1:** Results of Classified Patients of the Gastric Cancer based on the Four Criteria of Evaluation

	performance measure			
Method	F-score	Precision	Recall	Accuracy
k-NN	87.17	89.47	85	87.6
NB	87.99	84.61	91.66	87.6
DT	88.37	87.69	89.06	87.89
SVM	91.99	90.78	93.24	90.08

**Table 2:** Results of SVM Classification based on the Confusion Matrix Criterion

	Predicted: Healthy	Predicted: Cancer
Actual: Healthy	57	1
Actual: Cancer	3	61

**Table 3:** Results of DT Classification based on the Confusion Matrix Criterion

	Predicted: Healthy	Predicted: Cancer
Actual: Healthy	51	5
Actual: Cancer	9	57

**Table 4:** Results of the Bayesian Model Classification based on the Confusion Matrix Criterion

	Predicted: Healthy	Predicted: Cancer
Actual: Healthy	53	3
Actual: Cancer	7	59

**Table 5:** Results of the KNN Algorithm Classification based on the Confusion Matrix Criterion

	Predicted: Healthy	Predicted: Cancer
Actual: Healthy	49	8
Actual: Cancer	11	54

We selected 405 participants from the two groups of healthy and patient individuals who referred to Rajaee and Boalie hospitals. Due to the use of the data in these two hospitals, the sampling method was not used and the entire population was included in the study. Finally, out of a total of 1,550 patient records related to the study, about 405 records, which had complete data for analysis, were included in the study. A total of 11 characteristics existed in the databases that included the participants’ gender, age, weight loss, abdominal pain, Nausea, anorexia, dysphagia, Pernicious anemia, Melena, and abdominal mass. The data collection tool was a data extraction form designed based on the characteristics used for screening. To solve the class imbalance problem, resampling was applied. The results of this study were obtained based on the 10-fold cross validation technique. In this method, the whole database is initially divided into training and testing sets, then the training set is divided into 10 parts. In each repetition of the cross-validation process, one part is selected as the validation set, while the rest of data is selected as the training set. We should also note that 70 percent of the total database samples are determined as the training set and the remaining 30 percent are taken as the testing set. In this study, we used the machine learning methods because in a medical application, the patient’s medical information is usually accompanied with a very large number of factors (characteristics) and considering all these characteristics by the physician while deciding about the patient’s condition is difficult. In addition, application of the mathematical methods may be associated with error and complexity, which therefore results in low efficiency. Due to the large number of characteristics in gastric cancer patients, this issue has a particular importance. For this reason, we studied the four learning methods of SVM, DT, naive Bayesian model, and KNN to classify the patients with gastric cancer. Our aim was to determine which of these four machine learning methods has the highest precision in classifying the gastric cancer samples. The evaluation criteria to investigate the model on the database of patients with gastric cancer included Recall, Precision, F-score, and Accuracy. Data were analyzed using MATLAB^®^ software, version 3.2 (Mathworks Inc., Natick, MA, USA).

## Results

we compared the results of all four classification methods based on the four criteria mentioned above (Table 1). Accordingly, the best results achieved from all criteria were related to the SVM algorithm with accuracy of 90.08 and precision of 90.78. The DT, Bayesian model, and KNN algorithms followed the SVM model respectively in classification of the patients with gastric cancer.

In the second stage of evaluation, we compared the results of all classification methods on the basis of the Confusion Matrix (CM) criterion and the results are presented in Tables 2 to 5. The total number of test samples was 122, from which 62 samples were from the cancer class and 60 were derived from the healthy class. Based on these findings, the SVM algorithm had the highest rate of precision in classifying the samples. In the SVM algorithm, the number of healthy people who were diagnosed correctly was 57 cases and the number of cancer patients identified correctly was 61 (Table 2).

In the DT algorithm, the number of healthy individuals who were diagnosed correctly was 51 cases and the number of cancer patients diagnosed correctly was 57 (Table 3).

In the Bayesian model algorithm, the number of healthy people who were diagnosed correctly was 53 cases and the number of cancer patients diagnosed correctly was 59 (Table 4).

In the KNN algorithm, the number of healthy people who were diagnosed correctly was 49 cases and the number of cancer patients diagnosed correctly was 54 (Table 5).

Based on the results, the SVM algorithm classified the samples with higher precision than other methods. This model obtained the best results in classifying the samples regarding all criteria because of its high generalizability and flexibility in learning various issues. It should be noted that the DT algorithm was ranked second in the classification due to its dependence on educational data and less generalizability than the SVM. The Bayesian method assumes that the classification characteristics are independence, therefore the quality from each attribute is independent of each other. However, in many cases, one feature alone does not have any effect on the classifications of samples and provides better results when combined with other characteristics. The KNN algorithm had the weakest results since it does not use the learning technique, classifies samples only based on the distance criterion, and is sensitive to noise. 

## Discussion

In this study, the gastric cancer patients were classified using four machine learning algorithms. Considering the fact that medical databases consist of numerous characteristics naturally, it is very difficult for the physician to take into account all the characteristics and make decision about the patient’s condition. This also increases the probability of error in decision making about the patient’s condition. In such a situation, application of an automated method that includes problem learning and problem generalization to other conditions seems desirable. In medical databases, an individual may have many similarities with several classes; so, a method with appropriate generalizability is required. In order to solve the above problems, we suggested four methods of SVM, DT, naive Bayesian model, and KNN to classify the patients with gastric cancer in this study. Based on the results, the SVM had the best results in classifying the samples based on the mentioned evaluation criteria and in comparison with other methods of machine learning. In line with the results of the study conducted by Wang et al. in the Chinese treatment centers and based on the evaluation criteria, we found that the SVM algorithm was a suitable method for diagnosis of gastric cancer. In addition, the precision of SVM classification was very high (90.78), especially among the four types of classification, which indicates the potential application of the SVM model in diagnosing cancer (Wang and Huang, 2011).

However, Mahmoodi et al., (2017) studied one treatment center in Tabriz and reported that the DT algorithm was a suitable method for identifying the factors that influence the incidence of gastric cancer. The precision of the DT algorithm in predicting the gastric cancer was 85.56, which was very high. The findings reported by Silvera et al., (2014) also indicated that the DT method was applicable in predicting the effective factors in the incidence of gastric cancer and gastric reflux was the most important cause of gastric cancer. On the other hand, Kirshners et al., (2015) presented a multi-layered method, in which the clustering techniques and DT algorithm were combined. They provided a system for diagnosing and predicting the risk of gastric cancer. The results of DT algorithm, in their study indicated that individuals’ gender, occupational hazards, weight loss, family history, alcohol consumption, abdominal pain and melena were among the causes and symptoms of the gastric cancer. The precision criterion for using the SVM algorithm in the present study was almost 91percent. In this regard, Ahmadzadeh and Fiyuzi, (2013) showed that the proposed combinational system managed to achieve the precision of 85.8 percent by relying on database properties and by applying combination and interaction among different methods. Meanwhile, the mentioned methods are costly and time-consuming in spite of their high precision. Finally, although the SVM method has been used as an appropriate and valid algorithm for identifying effective factors in different studies (Chang et al., 2003; Jumutc et al., 2011; Wang, 2005; Zhang et al., 2018), it still has several advantages and disadvantages (Abe, 2005; Guyon et al., 2002; Yoo et al., 2012). One of the most important advantages of SVM, as a classification method, is the improvement of efficiency with the increase of data dimension. It is also relatively simple to teach and unlike the neural networks, is not stuck in the local maximum. This method has the ability to solve complex classification problems containing many layers and low traing samples. Regarding the disadvantages of this method, we can note that it requires a good kernel function and an appropriate parameter C. The computational complexity is also high in this method (Moulin et al., 2004). According to the mentioned evaluation criteria and in comparison with the other machine learning methods, the SVM provided the best results in classifying the samples. In order to continue the research in this realm and to improve the classification results, we can combine the feature selection methods with the proposed method. In addition, we can study the feature selection operations using the evolutionary methods and investigate their impact on the classification of patients with cancer. Since the samples of a medical database are not balanced, we can study the balancing method of the data collected from patients with gastric cancer and its effect on improving the classification results of the four classifying methods mentioned in this study. In addition, we can apply the evolutionary methods to find the optimal values of the SVM algorithm to reduce its computational complexity.
